# Supplementary material for: Multiplex qPCR discriminates variants of concern to enhance global surveillance of SARS-CoV-2
Source: PLoS Biol. 2021 May 7;19(5):e3001236. doi: 10.1371/journal.pbio.3001236 (PMC8133773; doi:10.1371/journal.pbio.3001236)
Supplement: S1 Table — SARS-CoV-2, Severe Acute Respiratory Syndrome Coronavirus 2. (DOCX) [file pbio.3001236.s001.docx]

| **Clade** | **Total sequences** | **ORF1a:**  **Δ3675-3677** | **Spike:**  **Δ69-70** | **% ORF1a:**  **Δ3675-3677** | **% Spike:**  **Δ69-70** |
| --- | --- | --- | --- | --- | --- |
| 19A | 16,034 | 5 | 9 | 0.0% | 0.1% |
| 19B | 8,869 | 1 | 4 | 0.0% | 0.0% |
| 20A | 81,139 | 22 | 6,494 | 0.0% | 8.0% |
| 20A.EU2 | 10,380 | 5 | 13 | 0.0% | 0.1% |
| 20B | 95,315 | 21 | 1,366 | 0.0% | 1.4% |
| 20C | 45,041 | 43 | 274 | 0.1% | 0.6% |
| 20D | 4,804 | 0 | 4 | 0.0% | 0.1% |
| 20E.EU1 | 91,540 | 2 | 7 | 0.0% | 0.0% |
| 20F | 12,613 | 4 | 0 | 0.0% | 0.0% |
| 20G | 11,276 | 0 | 22 | 0.0% | 0.2% |
| B.1.351 | 582 | 474 | 0 | 81.4% | 0.0% |
| B.1.1.7 | 25,262 | 25,178 | 25,142 | 99.7% | 99.5% |
| P.1 | 34 | 34 | 0 | 100.0% | 0.0% |
