# Supplementary material for: Multiplex qPCR discriminates variants of concern to enhance global surveillance of SARS-CoV-2
Source: PLoS Biol. 2021 May 7;19(5):e3001236. doi: 10.1371/journal.pbio.3001236 (PMC8133773; doi:10.1371/journal.pbio.3001236)
Supplement: S2 Table — RT-qPCR, reverse transcription quantitative PCR. (DOCX) [file pbio.3001236.s002.docx]

| **Set name** | **Nt positions** | **TM** | **Primer/probe** | **Sequence** |
| --- | --- | --- | --- | --- |
| CDC  N1 | 28,287-28,306 | 53.6 | Fwd primer | GACCCCAAAATCAGCGAAAT |
|  | 28,335-28,358 | 57.7 | Rev primer | TCTGGTTACTGCCAGTTGAATCTG |
|  | 28,309-28.332 | 63.3 | Probe | **FAM**-ACCCCGCATTACGTTTGGTGGACC-**BHQ1** |
| Yale  ORF1a  Δ3675-3677 | 11,229-11,248 | 60 | Fwd primer | TGCCTGCTAGTTGGGTGATG |
|  | 11,332-11,356 | 57.8 | Rev primer | TGCTGTCATAAGGATTAGTAACACT |
|  | 11,283-11,312 | 61.9 | Probe | **Cy5**-GTTTGTCTGGTTTTAAGCTAAAAGACTGTG-**BHQ2** |
| Yale  Spike  Δ69-70 | 21,710-21,733 | 59.3 | Fwd primer | TCAACTCAGGACTTGTTCTTACCT |
|  | 21,796-21,817 | 57.4 | Rev primer | TGGTAGGACAGGGTTATCAAAC |
|  | 21,755-21,779 | 61.2 | Probe | **HEX**-TTCCATGCTATACATGTCTCTGGGA**-BHQ1** |
