# Supplementary material for: Multiplex qPCR discriminates variants of concern to enhance global surveillance of SARS-CoV-2
Source: PLoS Biol. 2021 May 7;19(5):e3001236. doi: 10.1371/journal.pbio.3001236 (PMC8133773; doi:10.1371/journal.pbio.3001236)
Supplement: S3 Table — RT-qPCR, reverse transcription quantitative PCR. (DOCX) [file pbio.3001236.s003.docx]

| **Result** | **N1-FAM** | **Orf1a-Cy5** | **Spike-HEX** |
| --- | --- | --- | --- |
| Potentially B.1.1.7 (VOC) or B.1.525 (VOI) | CT ≤ 35 | Undetected | Undetected |
| Potentially B.1.351 (VOC), P.1 (VOC), or B.1.526 (VOI) | CT ≤ 35 | Undetected | CT ≤ 35 |
| Potentially B.1.375 | CT ≤ 35 | CT ≤ 35 | Undetected |
| Other lineages | CT ≤ 35 | CT ≤ 35 | CT ≤ 35 |
| Inconclusive | CT > 35 or undetected | Any value | Any value |

VOC, variant of concern. Thresholds need to be determined when using different RT-qPCR reagents or instruments.
