# Supplementary material for: Multiplex qPCR discriminates variants of concern to enhance global surveillance of SARS-CoV-2
Source: PLoS Biol. 2021 May 7;19(5):e3001236. doi: 10.1371/journal.pbio.3001236 (PMC8133773; doi:10.1371/journal.pbio.3001236)
Supplement: Attachment — Submitted filename: Rebuttal.pdf [file pbio.3001236.s006.pdf]

Editors

Thank you very much for submitting your manuscript "PCR assay to enhance global surveillance for SARS-CoV-2 variants of concern" for consideration as a Research Article by PLOS Biology. As with all papers reviewed by the journal, yours was evaluated by the PLOS Biology editors as well as by an Academic Editor with relevant expertise and by independent reviewers. The reviewers appreciated the attention to an important topic.

Based on the reviews, we will probably accept this manuscript for publication, provided you satisfactorily address any points raised by the reviewers.

We suggest a change of title in order to make it a bit more declarative, but please feel free to modify it: "Multiplex qPCR discriminates variants of concern to enhance global surveillance of SARS-CoV-2"

As you address these items, please take this last chance to review your reference list to ensure that it is complete and correct. If you have cited papers that have been retracted, please include the rationale for doing so in the manuscript text, or remove these references and replace them with relevant current references. Any changes to the reference list should be mentioned in the cover letter that accompanies your revised manuscript.

We expect to receive your revised manuscript within two weeks.

We thank the editors for their positive response and feedback. We have provided a point-by-point response to the comments raised by the reviewers. As specifically indicated by the editors, **we have now changed the title of our manuscript to “Multiplex qPCR discriminates variants of concern to enhance global surveillance of SARS-CoV-2”**.

Reviewer #1

Reviewer #1: "PCR assay to enhance global surveillance for SARS-CoV-2 variants of concern," by Vogels et al describes development and testing of a multiplex qPCR screening approach to identify probable SARS2 variant of concern samples. While the late 2020 discovery that S gene dropout PCR tests could indicate B117 variant infection was fortuitous, those results were far from definitive and not capable of identifying all variants of concern. By targeting an Orf1a deletion in addition to the spike deletion, a more targeted and broadly useful assay can be performed while using the same sample types and overall technology. The benefits and limitations of this assay are clearly described and the sample testing was robust. As a new reviewer of this revised manuscript I am not able to see the previous reviews to assess how they were addressed; however; I have no concerns with this work and encourage speedy publication.

We thank the reviewer for their time and kind words. We are happy to hear that the reviewer encourages speedy publication of our manuscript.

Reviewer #2

Reviewer #2: Tracking the emergence and spread of SARS-CoV2 variants has great public health importance, and the paper by Vogels and colleagues describes a real-time PCR-based approach to identify key variants of interest/concern; particularly B.1.1.7, B.135 and P.1. The authors state that such an approach would be of significant benefit where access to NGS analysis was limited, and even in resource-rich regions, where NGS was readily available, such an approach could be used to effectively triage samples for sequencing.

The approach take was robust and the validation, albeit on a relatively small number of clincial samples (especially samples from Brazil and South Africa), convincing. Whilst the methods were able to identify target variants, the discriminatory power was less than 90% for some variants, meaning that downstream sequence validation would be required. Similarly, the authors also state that the multi-plex PCR would not be able to replace existing diagnostic approaches, due to non-specific auto-fluourescence. Therefore, this method would be useful as an additional step between clinical diagnosis and downstream sequencing but would not be able to replace either.

It was unclear to me why this paper would be suitable for publication in PLoS Biology - it would be much more suited to a journal specialising in viral diagnostics.

We thank the reviewer for their time to review our manuscript and for the positive feedback. Our assay indeed provides a rapid screening assay for SARS-CoV-2 variants of concern, which complements clinical diagnostics and next-generation sequencing. While the manuscript was under review, **we have now screened a total of 1,361 samples with our assay, and have updated the manuscript with this new data.** Importantly, 100% of the B.1.1.7, B.1.351, and P.1 variant PCR assay results were in agreement with NGS data.

We have submitted our manuscript to PLOS Biology because it is an open-access journal that reaches a broad audience. Our assay is relevant for research/clinical diagnostic/public health labs with backgrounds in various fields, and therefore we believe PLOS Biology is an excellent target journal for our manuscript.
